# Cooperativity Dominates the Genomic Organization of p53-Response Elements: A Mechanistic View

**DOI:** 10.1371/journal.pcbi.1000448

**Published:** 2009-07-24

**Authors:** Yongping Pan, Ruth Nussinov

**Affiliations:** 1 Basic Research Program, SAIC-Frederick, Inc., Center for Cancer Research Nanobiology Program, NCI-Frederick, Frederick, Maryland, United States of America; 2 Sackler Institute of Molecular Medicine, Department of Human Genetics and Molecular Medicine, Sackler School of Medicine, Tel Aviv University, Tel Aviv, Israel; Duke University, United States of America

## Abstract

p53-response elements (p53-REs) are organized as two repeats of a palindromic DNA segment spaced by 0 to 20 base pairs (bp). Several experiments indicate that in the vast majority of the human p53-REs there are no spacers between the two repeats; those with spacers, particularly with sizes beyond two nucleotides, are rare. This raises the question of what it indicates about the factors determining the p53-RE genomic organization. Clearly, given the double helical DNA conformation, the orientation of two p53 core domain dimers with respect to each other will vary depending on the spacer size: a small spacer of 0 to 2 bps will lead to the closest p53 dimer-dimer orientation; a 10-bp spacer will locate the p53 dimers on the same DNA face but necessitate DNA looping; while a 5-bp spacer will position the p53 dimers on opposite DNA faces. Here, via conformational analysis we show that when there are 0–2 bp spacers, p53-DNA binding is cooperative; however, cooperativity is greatly diminished when there are spacers with sizes beyond 2 bp. Cooperative binding is broadly recognized to be crucial for biological processes, including transcriptional regulation. Our results clearly indicate that cooperativity of the p53-DNA association dominates the genomic organization of the p53-REs, raising questions of the structural organization and functional roles of p53-REs with larger spacers. We further propose that a dynamic landscape scenario of p53 and p53-REs can better explain the selectivity of the degenerate p53-REs. Our conclusions bear on the evolutionary preference of the p53-RE organization and as such, are expected to have broad implications to other multimeric transcription factor response element organization.

## Author Summary

p53-response elements (p53-REs) are 20 base pair DNA segments embedded in the genome that are able to bind anti-tumor protein p53 and trigger biological functions such as DNA repair or self-destruction of the cell. These functions are modulated through selective binding of p53 to degenerate p53-REs. Understanding how the cells choose p53-REs for enacting a specific biological function is crucial for obtaining insight into cancer development. Experimental data indicate that the majority of p53-REs contain a small intervening spacer in the middle of their p53-REs. Here, we propose that there is a relationship between the organization of the p53-REs and binding cooperativity. To test this hypothesis, analysis of existing crystal structures and modeling of p53-DNA complexes was undertaken. The outcome shows that when there are 0–2 base pair spacers, there are more interactions between the p53 subunits, and the p53-DNA binding is cooperative. When the spacer sizes are larger, the interactions among p53 subunits are diminished. Our results indicate that cooperativity of the p53-DNA association dominates the genomic organization of the p53-REs and suggest that different mechanisms of activation may be at play for those p53-REs with large spacers.

## Introduction

Tumor suppressor p53 protects the genome through specific and cooperative interactions with p53-response elements (p53-REs) [1]–[3]. p53-REs consist of two 10-base pair (bp) palindromes with the sequence of 5′-PuPuPuC(A/T)(A/T)GPyPyPy-3′ for each palindrome, where Pu and Py stand for purine and pyrimidine bases, respectively [4]–[6]. While the two half sites can be separated by as many as 20 bps [5],[7],[8], experimental data derived from different ChIP-based techniques indicated that an overwhelming majority (>80% from the human genome and 74% from yeast) have 0–2 bp insertions [7], [9]–[11]. This extremely skewed distribution of the p53-RE organization raises the question as to why the genome preferred small spacers; whether those with large spacers can bind p53 cooperatively; and if not, how they are functionally relevant.

The p53 protein structures have been well studied [12]. p53 is a tetramer of four homologous peptide chains, each of which consists of the N-terminal domain which regulates the p53 transactivation activity [13], the specific DNA-binding core domain [14], the tetramerization domain [15], and the C terminal domain. Crystal and NMR structures revealed the specific p53 core domain-DNA binding mode. Molecular modeling and simulations of p53-REs without bp insertions suggested that the association of the four p53 DNA-binding domains with each quarter site on the 20-bp DNA duplex present a conformation in which the two p53-domain dimers were in close contact [16],[17]. Since cooperativity was observed *only* when the full p53-binding site was present for both the full p53 protein and the core domain so that two p53 core domain dimers can bind simultaneously [18]–[20], it is believed that the p53 core domain dimer-dimer interactions are largely responsible for the cooperativity [18], [21]–[23]. In addition, the impact of each nucleotide within the p53-RE on the binding affinity was also well studied [24]. However, how the tetrameric p53 protein binds cooperatively to other p53-REs with base pair insertions, and in particular whether the p53 core domain dimer-dimer interactions play a role in cooperative binding in these cases, is not known. It was observed however, that binding of p53 to p53-REs with 10 bp insertions had moderate affinity/cooperativity while binding to those with 5-bp insertions was the weakest [20]. A more recent study on the relationship between spacer size and binding affinity or transcriptional activity reveals that the binding affinity decreases sharply with small increase in spacer size [25]. Such a correlation was also observed in the binding analysis for consensus estrogen response elements which also contains a palindromic sequence of 6-bp in each half site [26]. Modeling the p53 with different p53-REs is important to the understanding of cooperative binding, the structural and the genomic organization of the p53-REs.

Recently there was significant progress in the structural biology of p53 [27]–[32]. The direct determination of the tetrameric p53 core domain-DNA complex structure and its variants validate the specific p53 core domain-DNA complex organization. The structural variability observed among the different crystal and NMR structures, and in modeling suggests that p53 dimer-dimer interfaces and the DNA conformations are polymorphic, depending on the DNA sequence and spacer size between the p53-REs half sites. These structural and biophysical data and modeling results [16],[17],[32],[33] pave the way for modeling p53-DNA complexes with different bp insertions and for exploring putative dimer-dimer interfaces responsible for cooperative DNA binding. The validity and capability of molecular dynamics simulations and modeling in reproducing and predicting structural properties of protein-nucleic acid complexes have been reviewed recently [34]. Here, we have constructed and simulated p53-p53RE models with variable spacers to look into a relationship between spacer sizes and cooperativity. Previously, fluorescence anisotropy and ultracentrifugation studies of 20 p53-REs without spacers from a broad range of genes provided direct evidence that cooperativity plays a key role in p53-DNA recognition [23]. Consistently, our results lead us to conclude that the overwhelming preference for genomic p53-REs with no or with small spacers between the two half sites in the human genome manifest the crucial role of cooperativity in p53-DNA interactions. *Cooperativity implies efficient regulation*, *thus dominating p53-RE genomic organization.* Efforts are being made to predict DNA recognition sites [35],[36]; findings such as those presented here are expected to assist in such undertakings.

## Results

### Simulation of tetrameric p53-DNA crystal structure

The tetrameric p53-DNA crystal structure solved by the Shakked group revealed a unique p53 dimer-dimer organization [30] that is different from the previously modeled structure with no spacer [16],[17]. However, the crystal structure is actually a pseudo p53 core domain-DNA complex, i.e. two p53 dimer-DNA motifs stacked together [30]; thus, it is essential to probe the stability of this conformation in solution to see if or how the conformation will change when the two DNA segments are covalently linked, and whether the p53 core domain dimer-dimer interface is relevant to cooperative binding. Two constructs derived from the crystal structure were built and simulated: the original crystal structure (‘Xtal’, conformation I, pdb file 2AC0) and with the two DNA fragments covalently linked (‘Link’) without any other structural or conformational modifications.

**Figure 1:**
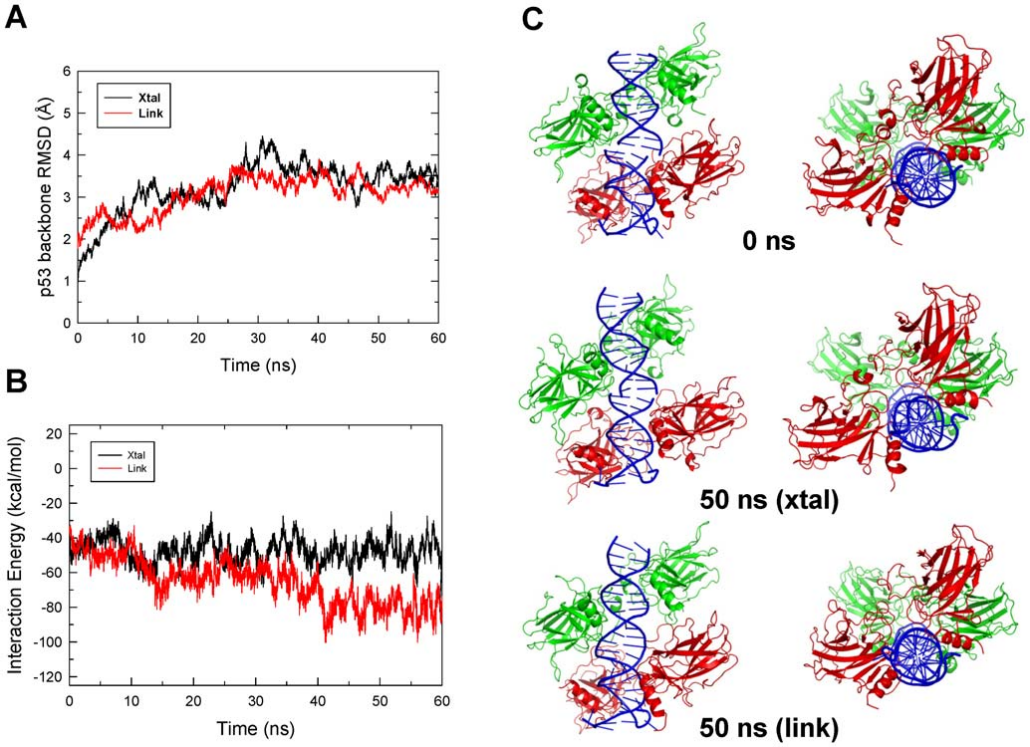
Structural changes of three Shakked's crystal structure-based p53 core domain-DNA complexes during the 60-ns molecular dynamics simulations. (A) Cα-RMSD of the p53 tetramer for the crystal structure (‘Xtal’) and of the crystal structure with DNA linked (‘Link’). (B) p53 core domain dimer-dimer interaction energy changes in the simulations for the crystal structure and the crystal structure with the DNA linked. (C) Comparison of the conformations of the starting crystal structure, snapshot at 50 ns from the simulations of the crystal structure and the crystal structure with the DNA segments linked.

The average root-mean-square difference (RMSD) of the p53-tetramer backbone was moderate (3.4 Å for the last 20 ns, Fig 1A) and the dimer-dimer interaction energy did not change much from the initial structure (Fig 1B) for the crystal structure simulation. However, the p53 dimer-dimer organization was not well maintained, with one side of the dimer-dimer interactions lost (Fig. 1C), possibly due to the altered stacking of the two DNA segments (Fig 1C). For the ‘Link’ simulation (the two DNA segments covalently linked), the overall RMSD was similar to that of the ‘Xtal’ simulation (average 3.1 Å for the last 20 ns, Fig 1A). However, the interactions between the two p53 core domain dimers increased significantly (Fig 1B). The DNA conformation at the linked region was very different from the initial structure at the end of the simulation (compare Figure 1C top and bottom panels) and was indistinguishable from the rest of the DNA segment (Fig 1C). The increased p53 dimer-dimer interaction at the interface was likely facilitated by the shortened distance between the two DNA segments after the covalent linkage which brought the two dimers closer. In addition, the C1 atoms' distance between two adjacent bases from opposite chains in the initial structure was almost 5 Å longer than the typical B-form DNA distance [37] (16.7 Å vs 12.06±0.57 here, with a span of 10.55–12.92). It should be noted that the DNA conformation in the crystal structure simulation was similar to the “Link” conformation. This result suggests that the observed DNA stacking mode is the preferred way for base pairs to interact, regardless of the covalent linkage.

### Conformational search of the dimer-dimer interface

**Figure 2:**
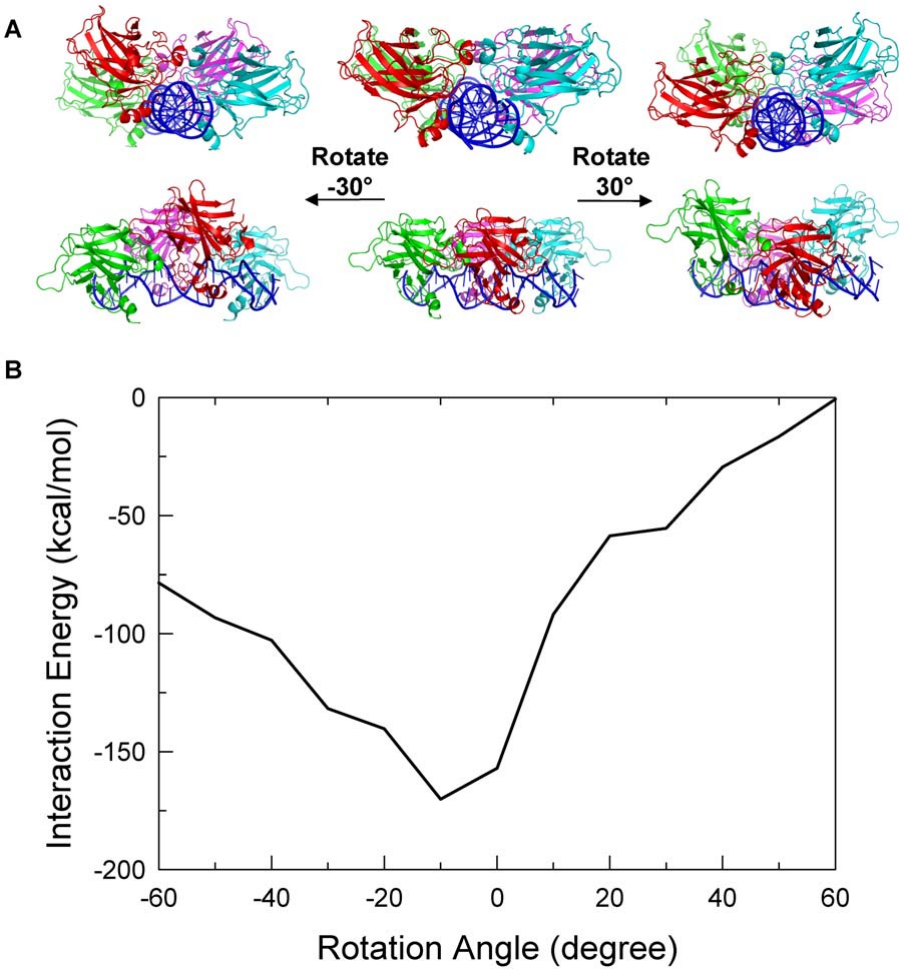
p53 dimer-dimer interaction energies for various dimer-dimer organizations derived by rotating one p53 dimer with respect to the other in the tetrameric p53-DNA complex. (A) Illustration of the relationship between the rotation angle and the change of the p53 dimer-dimer organization. The rotation angle was defined as 0 when the two dimers were aligned. Clockwise rotation of the p53 dimer at the front (red and cyan) resulted in a positive angle and a negative angle otherwise. (B) The p53 dimer-dimer interaction energy changes upon the changes of the dimer-dimer organization. Rotation angles beyond the range shown in the plot were not presented due to unrealistic twisting of the DNA. The most favorable organization based on the dimer-dimer interaction energy was the one with a rotation angle near 0.

p53 dimers bound to the p53-REs without spacers are most likely to have significant interactions with each other due to their closeness. Experiment and simulations have shown that p53 dimer-dimer interactions can occur through different DNA-bound organizations, such as staggered with an clockwise rotation angle of about 30 degrees between the two p53 dimers [30], or with anti-clockwise rotation angle of 10–20 degrees [16],[17]. Given DNA flexibility and the p53 dimer-dimer interaction modes such as those above, it is likely that p53 core domain dimers interact with different organizations under different conditions. However, functional organizations should be energetically most favorable and therefore, it is essential to obtain the preferred dimer-dimer interactions in the biologically relevant states. To find the most favorable interactions, the rotational conformational space of the p53 dimers with respect to each other on the DNA was searched by rotating half of the p53 tetramer-DNA complex dissected at the center of the p53-REs. The starting conformation was derived from the Shakked crystal structure by removing the two terminal base pairs and aligning the two p53 dimer-DNA motifs (see Methods). The starting structure and the dimer-dimer interaction energy versus the rotation angles between them are shown in Figure 2 (A middle panel and B, respectively). The most favorable dimer-dimer interactions had the two dimers eclipsed or slightly rotated from each other, with a rotation angle between 0 and 15 degrees (Fig 2), consistent with the conformation observed in previous MD simulations [17]. Since DNA takes 10–11 base pairs (for B and A forms respectively) for a complete helical turn or 16.4–18 degrees rotation every half bp, the fact that the two p53 dimers were half bp away from an eclipsed arrangement on the DNA is in accordance with the preferred canonical DNA conformations.

It should be noted that a dimer-dimer rotation by 30 degrees yielded an organization similar to Shakked's crystal structure (Figs 1C and 2A) except that there was no bp spacer in the binding site. In this conformation, the DNA bent only slightly toward the p53 (wrapped around p53, Fig 2A, left panel) while the DNA bent away from the p53 complex (or being wrapped by p53) when rotated in the opposite direction (Fig 2A, right panel). Although the bending extent was not as significant in either direction as observed in the MD simulations due to the nature of the energy minimization, the results show that the DNA was flexible and played a role in cooperative binding.

### Modeling of p53 dimer-dimer interfaces for complexes with base pair insertions

#### Model construction

Figure S1 shows the tetrameric p53-DNA models using straight DNA duplex segments with 1–10 bp insertions. Since it takes 10–11 bps for a complete helical turn, the addition of each bp increases the angle between the two dimers by roughly 36 degrees. The models illustrate that an addition of an extra base pair in the spacer changes dramatically the relative orientation and distance between the two dimers. For complexes with 3–8 bp insertions the two p53 dimers are at least 108 degree apart in terms of the rotation angle and therefore are difficult to render any dimer-dimer interactions. For 9 and 10 bp insertions it is possible for the dimers to interact when bound to a pre-bent DNA conformation since the two dimers would be facing the same side of the DNA (details in Methods). Thus, MD simulations were performed on complexes only with insertions of 1, 2, 9 and 10 bps. The relationship between the bp insertion size and the dimer-dimer interaction should help delineate the reasons why the genomic organization of the p53-REs has a preferred distribution.

#### MD simulations

**Figure 3:**
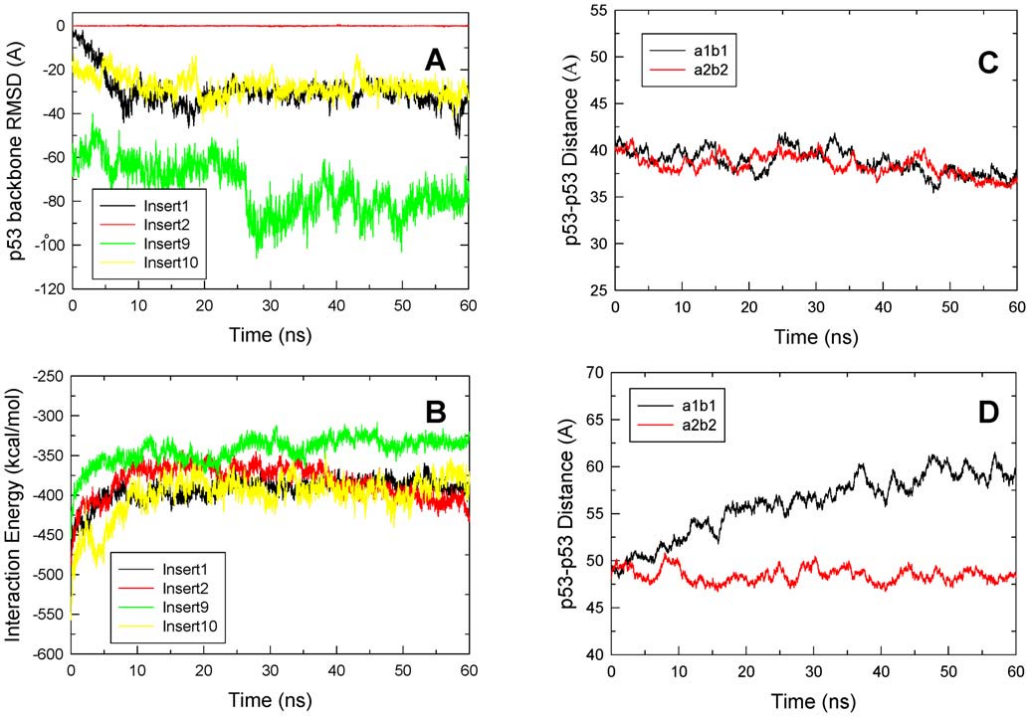
Structural and energy properties of the four complexes with spacer sizes of 1, 2, 9 and 10 base pairs. (A) and (B) p53 dimer-dimer interaction energy and p53-DNA interaction energy, respectively. (C) and (D) the distance between the centers mass for the two pairs of p53 core domain for one and ten bp insertion complexes, respectively. The interacting p53 core domain pairs were labeled in Figure 4.

**Figure 4:**
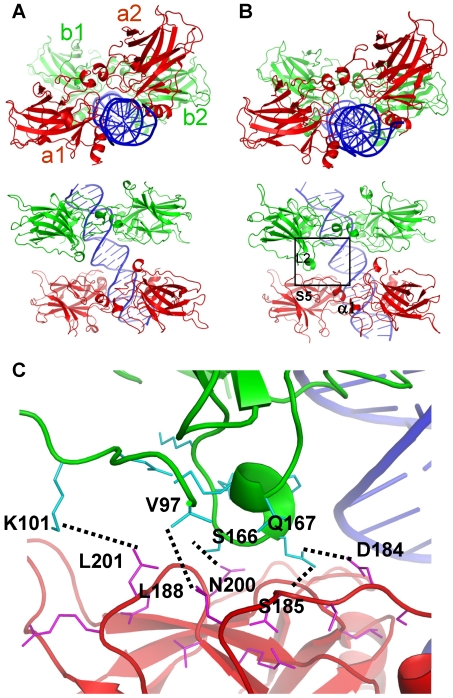
p53 core domain tetramer-DNA complex model with 1-bp insertion. (A) Starting structure conformation with four p53 subunits labeled a1, b1, a2 and b2, respectively and (B) average structure of the last 5 ns trajectory (55–60 ns) with two views for each structure. The motifs involved in the interactions at the interface including loop L2 (shown in magenta) from one subunit and those residues between strand S5 and α-helix 1 (colored cyan) in the other were highlighted. (C) Atomic details of the interactions at the dimer-dimer interface. The backbone of the core domains were colored same as in (B). Residues that are within 7 angstroms of the other domains were shown in thin sticks. Interacting residues were related with dotted lines.

Figures 3A and 3B show the interaction energy changes of the four models during the course of 60-ns MD simulations. For 1-bp insertion, the interaction energy reached equilibration after 20 ns and the final structure along with the starting one are shown in Figure 4. The dimers moved closer toward each other as further confirmed by the slightly shortened distances (Fig 3C). Inspection of the dimer-dimer interface revealed shape complementarity between loop L2 from one subunit (Fig 4B, colored in magenta) and strand S5 and the C terminal loop to helix α1 (Fig 4B, cyan) from the other subunit. In addition, three hydrogen bonding interactions and two hydrophobic contacts were observed at the optimized interface, providing the atomic basis for the enhanced interactions (Figure 4C). Since the dimers moved closer to each other, the DNA suffered some conformational change and bent toward the p53 tetramer (Fig 4B, left panel), suggesting the high adaptability of the DNA. Thus, for one-bp insertion, starting from straight DNA the system was able to obtain reasonable dimer-dimer interactions, forming the basis for cooperative binding of this particular response element organization. p53-REs with one-bp insertion were also significantly populated and were only second to the no spacer p53-REs [7]. The dimer-dimer contact stability in this complex suggests a role in cooperative binding.

For the two-bp insertion p53-REs, the dimers were rotated about 72 degrees away from each other and the shortest distance between them was large in the starting structure (Figure S2A). The 60-ns MD simulation did not yield any significant interaction between the two dimers (Fig 3A). The conformational change was small relative to the initial structure and DNA deformation or bending was not observed either (Figure S2). Although the simulation did not initiate from bent DNA, it appears that the contact would be minimal and may not be sustainable even if the simulation initiated from bent DNA given the relative orientation of the two dimers.

For 9- and 10-bp insertions, the distance between the two dimers would be very large if the dimers were to bind to straight DNA. The DNA must bend significantly for the dimers to interact. Here, a series of bent DNA conformers were generated and used to match the dimers onto p53-REs with 9- or 10-bp spacers. The conformers that had the most extensive dimer-dimer interactions were selected, energetically minimized, and subjected to MD simulations. Figures S3A and S3B illustrate the initial and final structures for the 9-bp insertion. Figure 3A shows the dimer-dimer interaction energy change. Unexpectedly, the dimer-dimer interaction energy was more favorable than those for the other three models with 1, 2 and 10 base pair insertions. Examination of the interface details revealed shape complementarity between the two dimers (Fig S3C). In addition, the residues made multiple salt bridges, hydrogen bonding and other interactions across the interface (Fig S3C). However, the interaction between the protein and the DNA was significantly reduced (Fig 3B) due to the relaxation of the DNA. Comparison between panels A and B in Figure S3 reveals that the bending extent of the DNA was reduced in the final structure due to the release of DNA stress. The overall extent of bending was 60.6 degrees in the starting conformation while in the average structure from 55 to 60 ns it was only 27.3 degrees. Thus the significance of the dimer-dimer interaction was offset by the loss of specific p53-DNA interactions. In order for the system to maintain the cooperative binding both specific p53-DNA interactions and p53 dimer-dimer contacts are required [38]. In the second 60-ns run of the simulation with slightly different starting conformation (Figure S4C), the results were very similar to the first run, with the p53 dimer-dimer interactions (Figure S4A) much stronger than in complexes with different spacer sizes (Figure S4B).

**Figure 5:**
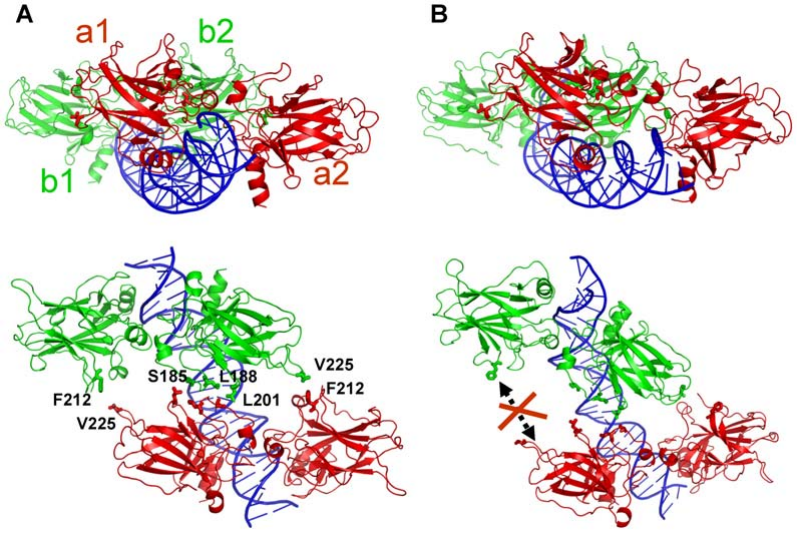
p53 core domain tetramer-DNA complex model with 10-bp insertion. (A) Starting structure conformation. (B) Final structure after the simulation (60 ns). Residues within 4.5 angstroms across the interface in the starting conformations are shown in sticks. In the final conformation, the monomer pair of the dimer interface lost their interactions. p53 subunits were labeled a1, b1, a2, and b2, respectively.

In the initial 10-bp spacer complex model, the two dimers were aligned on the same side of the DNA (Fig 5A). The conformation with the best dimer-dimer interactions had some hydrophobic contacts but lacked shape complementarity between the two dimers as compared to the model without insertions (Fig 5A). After the initial 20-ns relaxation, the dimer-dimer interaction energy was stabilized and the specific p53-DNA interactions were well maintained (Fig 3B). However, one pair of the p53 subunits gradually lost their contact with each other, although the other pair still interacted, suggesting that the initial constructed conformation is unstable. Figure 3D shows that the centers of mass distance for the disrupted pair increased by approximately 10 Å by the end of the simulation. Structural comparison showed that the dimers sled with respect to each other and new interactions were gained at the interface, compensating the lost interactions in the initial conformation. In order to confirm the instability of the conformation, a second simulation was performed on a slightly different starting conformation. The interaction energies, distance changes between the p53 core domains across the dimer-dimer interfaces, and the average structures are shown in Figure S5. Although the p53-DNA interactions were stabilized after 30 ns (Figure S5B), the p53 dimer-dimer interaction energy and the distances continued to become less favorable for the associations. Figure S5D clearly shows that one p53 dimer (red) sled away from the other (green), leading to the loss of the p53 dimer-dimer interactions. Thus, both simulations suggested that the p53 dimer-dimer interactions built in the initial model were not sustainable without the help of other factors.

To further characterize the stabilizing/destabilizing factors in these complexes, DNA conformational energies were calculated (Table 1). The differences in total energy between the starting and the final conformations reflect the DNA deformational strain or energy penalty to hold the DNA in the starting conformation. The data indicate that consistent with the bending extent, the complexes with the insertions 9 and 10 have much larger deformation energy than those with insertions 1 and 2 (Table 1). Comparison with the p53 dimer-dimer interactions in Figure 3A suggests that the stabilizing energy for insertion 10 was largely offset by the deformation energy of the DNA. For insertion 1, since there was little difference in DNA stress between the starting and final conformation, the p53 dimer-dimer interaction can in large part serve as the stabilizing factor. For insertion 9, despite the DNA deformation energy penalty, substantial stabilizing energy is still retained; however, as discussed earlier (Fig 3B) the large deformation energy resulted in certain DNA relaxation leading to larger disruption of specific p53-DNA interactions versus other complexes, thus questioning the functional relevance of this particular conformation.

**Table 1:** DNA energy difference between the starting and final conformations (kcal/mol)^a^.

model	Starting conformation (0–5 ns)			Final conformation (55–60 ns)			Difference		
	Total	Elec	VDW	Total	Elec	VDW	Total	Elec	VDW
Insert1	1460.7±23.2	−242.9±2.7	61.4±13.0	1454.2±23.5	−242.6±2.7	55.4±12.8	6.5	−0.3	−6.0
Insert2	1529.3±23.0	−252.8±3.0	61.0±13.8	1523.9±22.2	−254.5±3.3	63.6±12.7	5.4	1.8	−2.6
Insert9a	3241.7±38.1	−725.4±9.0	−8.0±19.7	3214.4±33.9	−731.0±6.2	−22.0±19.1	27.2	5.6	14.0
Insert9b	3247.7±41.5	−732.9±8.0	−0.1±20.0	3209.1±36.5	−735.9±5.7	−15.5±18.8	38.7	3.0	15.4
Insert10a	3366.7±40.5	−741.3±7.0	−3.6±21.0	3350.5±34.2	−747.3±5.7	−10.3±18.7	16.4	5.6	6.7
Insert10b	3375.2±50.8	−742.8±10.6	−4.1±22.2	3349.2±32.5	−748.7±5.6	−10.4±20.7	26.1	6.0	6.3

a Calculated energies were the arithmetical averages over 250 frames from the 5 ns trajectory segments. The first and the last 5 ns of each trajectory were used to estimate the energies for the starting and final structures, respectively. Calculation protocol was the same used for calculating interaction energies shown in Figures 1 and 3.

### Comparison of different p53 dimer-dimer interface models

**Figure 6:**
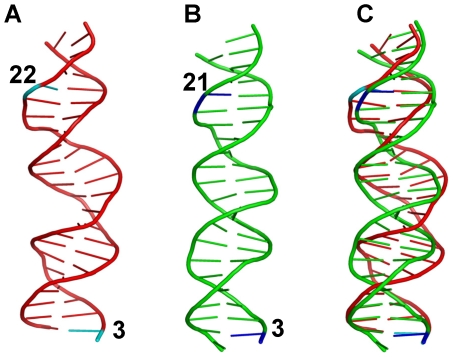
Comparison of the DNA structures from different simulations. (A) Average structure (55–60 ns) from the trajectory of Shakked's crystal structure with the DNA linked. (B) Average structure (55–60 ns) from the one base pair insertion complex simulation. (C) Superposition of (A) and (B). The positions for the two base pairs that were highlighted in cyan and blue were labeled. The superposition shows that the two bases (3 and 22) in the crystal structure overlapped well with the two bases (3 and 21) in the one base pair insertion complex. This figure shows the DNA unwinding and compression in conformation derived from the crystal structure simulation.

The results of the simulations of Shakked's crystal structure with the two DNA segments covalently linked suggest a stable complex with the two dimers interacting favorably (Fig 1C). This structure can be considered as a complex with a 2-bp insertion in the p53-RE. Unfortunately, simulations of straight DNA with 2-bp insertion did not yield a similar organization because of the starting B-form DNA conformation (Figure 3A). This suggests that if the two dimers first bind to straight canonical B-form DNA, then the interaction between the two dimers would not take place without the help of other factors to force the DNA conformational change. For the two dimers to interact, the DNA has to unwind to some extent to change the relative orientation of the two dimers. On the other hand, simulation with 1-bp insertion starting from straight DNA led to a conformation very similar to the simulated Shakked's crystal structure with the DNA linked (Figs 1 and 4). In both structures, there were two interacting regions across the dimer-dimer interface: the H2 helix interacting with the β-strand S7 and S8 loop with two hydrogen bonds between residues Ser163 and Gln164 from one monomer and Thr137 from the other. Other interactions were sparse. For the complex with two base pair insertions as in Shakked's crystal structure, not only do the two dimers have to rotate significantly with respect to each other, but the DNA duplex also has to compress along the helical axis in order to bring the two dimers together. The DNA end-to-end distances were measured for the three relevant simulations (Table 2). For models insert1 and insert2, the DNA lengths were very similar (65.6 and 65.2 Å respectively), suggesting very little or no compression of the DNA. However, in the “Link” model the DNA was 2.4 Å shorter than the insert1 model for the 20 base pair stretch (Table 2). Alignment of the DNA segments revealed that the 20-bp DNA segment from the “Link” simulation superimposed well with the 19-bp DNA segment from the insert1 simulation (Fig 6). This compression effectively shifted the DNA conformation away from the canonical B form. To further characterize the DNA conformational differences, global base pair X-displacement and inclination were calculated. As indicated previously [39], here too the inclination data were not as expected (data not shown), while the X-displacement points to a difference between the B-form DNA (models insertion1 and insertion2) and that of the compressed DNA (Link), with a value of ∼−0.8 versus −1.57 Å.

**Table 2:** Comparison of DNA end-to-end distances.

Model	Simulation Trajectory	DNA segment measured (bps)^a^	End-end distance (Å)	End-end distance per 20 bp (Å)
Crystal structure		22	70.5	64.1
Crystal “link”	45–50 ns	22	69.5±1.1	63.2
Insert1	55–60 ns	21	68.9±1.2	65.6
Insert2	55–60 ns	22	71.8±0.74	65.2

a The number of base pairs (bps) includes the two half sites and the spacer.

DNA is known to be able to inter-convert between the A and B forms under certain conditions; this allows segments with 2-bp insertion to form a complex with p53 with similar p53 dimer-dimer interactions as with one bp insertion. In the crystal structure, the DNA is obviously under-wound and compressed. The favorable interactions between the dimers and crystal packing provided additional energy source for the stability of the complex. The A/B DNA conversion allows p53 dimer-dimer interactions when they are bound to p53-REs with 1–2 bp insertion coinciding with the significant population of p53-REs with 2 base pair insertions [9]. Further study of their role in the p53 cooperative binding would be of great interest.

## Discussion

Putative p53-REs have been extensively characterized through different experimental and computational approaches [7], [9]–[11],[40],[41]. While the spacer size can consist of as many as 20 base pairs, the majority of the p53-REs in the human genome consist of 20 continuous bps without spacers. Further inspection of the p53-REs obtained from the human genome reveals that the most abundant among those with insertions contain a single bp spacer [7],[9]. Interestingly, the population of p53-REs with a 2 base pair spacer is only second to those without or with 1 base pair spacers [9]. Other than this extremely skewed distribution of the spacer size, the distribution of spacer sizes with 3–20 bps did not show any obvious pattern. These data prompted us to study the inter-relationship between cooperative interactions of the p53 core domain dimers and p53-DNA interaction, and the p53-RE occurrences with specific spacer sizes. Analysis of synthetic promoter libraries in yeast suggested that the combinations of cis-regulatory sites can be understood through protein-DNA and protein-protein interactions, highlighting the inter-relationship between the organization of the DNA sequence, cooperativity, and transcription [42]. The correlation between spacer size and the binding affinity/transcriptional activity [25] and the results of this work indicate that cooperative interactions between p53 dimers dominate the p53-RE organizations.

Cooperative complex formation is common in transcriptional activation, repression, DNA replication and recombination, and likely in all cellular processes. Cooperativity governs folding and regulation. Cooperativity in protein-DNA assemblies is widely recognized: if two (or more) proteins interact with the DNA and at the same time with each other, DNA binding is more favorable than the sum of the affinities of individual protein-DNA interactions [43],[44]. In the case of p53-DNA binding, the core and the tetramerization domains, and other regions of p53 contribute to the overall cooperativity. In p53 core domain dimerization the packing of helix H1 and loop L1 play an important role in cooperative DNA binding [45]. In the tetrameric p53 binding both the p53 core domain dimers interactions with DNA and with each other are the primary factors responsible for specific DNA binding and cooperativity. In this work, the tetramerization domain was not modeled into the organization of the complexes, since the interaction of this domain with the core domain is unknown; and in particular, available data indicate that this domain is unlikely to change the picture of the core domain dimer-dimer interactions. Further, fluorescence anisotropy data indicate that the full-length protein has similar DNA binding affinities as the core domain alone [23]. Our results clearly show that only those p53-REs with small spacers of up to 2 base pairs can elicit efficient p53 dimer-dimer interactions; large insertions with more than 3 bps would not involve direct cooperative dimer-dimer interaction.

However, a small number of putative p53-REs with large spacers do exist, possibly the outcome of the evolutionary selection process: it is known that some weak functional elements are highly conserved across species and these less “efficient” species are compatible with biological survival. Importantly, these larger-spacer p53-REs are more frequently observed in negatively regulated genes [46]. Therefore, it would be interesting to look at their p53 organization on the response element, and the structural basis of possible binding cooperativity. p53-REs with 5- or 6-bp insertions have the weakest binding even with full-length p53 [20]. To have direct dimer-dimer cooperative interactions in such systems, the DNA has to be extremely distorted; therefore it is unlikely. In a more likely scenario one dimer binds to one half-site specifically at any given time and the other p53 dimer binds non-specifically next to it. This may also apply to the case of 9-bp insertion: the two p53 dimers could initially bind specifically to their respective half sites; however, our results suggest that when the DNA-bound dimers come into contact with each other, they would lose some specific interactions. Alternatively, other transcriptional or regulatory factors join the three or more party-associations attaining the cooperative interactions. For insertions with 10 bps, cooperative p53-DNA interactions can still be mediated by p53-p53 interactions only. One possible scenario is the organization presented in this work although the interactions in the initial model were weak and not sustainable in the simulation. Another possibility is that two p53 core domain tetramers still bind shoulder-to-shoulder, but with one dimer pair from each tetramer binding specifically to the p53-RE half site, and the remaining pair from either tetramer binding to the intervening 10 base pair DNA non-specifically. p53 has been shown to form octamers upon binding to DNA, although the actual organization was proposed to be different. A shoulder-by-shoulder arrangement of two p53 tetramers can be reasonably stable, since the three putative p53 dimer-dimer interfaces would be similar due to the 10-bp spacing. This organization might be workable under extreme conditions at high cellular p53 concentrations. In any case, it is not surprising that these p53-REs have some functional role, since even a half site of DNA can have significant transcriptional activity [25], although with as yet unknown activation mechanisms. Additional differences between positive and negative regulation may involve post-binding events such as modification or recruitment of other transcriptional factors, although currently no clear data are available. Understanding the mechanisms associated with different p53-REs will not only illuminate events related to transcription regulation and cellular switches, but also evolutionary processes [46].

DNA is flexible; in simulations of a straight DNA model with no bp insertions between the half sites, the DNA bent significantly away from the protein. In this no-insertion case, the p53 dimers make favorable contacts, resulting in an optimized p53-DNA complex in which one p53 dimer rotated slightly with respect to the other [17]. In the case of one bp insertion, the DNA bent in the opposite direction, with one dimer rotated clockwise with respect to the other. In a p53-RE with two bp insertion the DNA may unwind to allow favorable p53 dimer-dimer interactions. With insertions of 9 or 10 bps, the DNA would have to bend significantly to allow the two p53 dimers to reach each other. Clearly, DNA conformational changes play important roles in specific p53-DNA binding and cooperative interactions. A recent work emphasized DNA allostery [47] and consequently, cooperativity. Allosteric effects are involved in all perturbation events of dynamic biological macromolecules, DNA, RNA and proteins [48]–[51]. And *all* allosteric effects are cooperative.

While the organizations of the p53-REs are increasingly understood, the mechanism for its selective activation is not known. Two models were proposed: the selective binding model and the selective context model [52]. The selective binding model highlights the importance of binding affinity and architectural specificity while the selective context model postulates that the post-binding events such as recruitment of other transcriptional factors determine selectivity. Mechanistically, selective binding relates to conformational selection [53]–[58]; a favored p53 population would bind a consensus p53-RE DNA with high affinity. On the other hand, p53-REs with different sequences present different distributions of the DNA conformational ensembles [59]; these would selectively bind to different p53 conformers. Low populations of these p53 conformers could increase via allosteric binding events to other protein factors whose concentrations rise under certain (stress, certain cell cycle stage, DNA instability, etc) conditions. Such a p53 dynamic landscape scenario can better explain the selectivity of the degenerate genomic p53-REs. In this regard, it is intriguing that post-transcriptional modifications, such as the phosphorylation of serine 46, can promote the activation of apoptosis while the mutant S46A p53 only triggers cell cycle arrest, thus altering the activation target [60]. Phosphorylation can lead to changes in protein activity through three major ways [61]: via direct interference, with the phosphate group blocking the substrate binding site; through formation of binding-competent sites; or most commonly, by a conformational change, with the phosphate group acting as an allosteric effector through conformational perturbation [51], [62]–[64]. The altered, now more populated conformational state of the p53 could favor an altered p53-RE. In this regard, the recent results of Riley et al. related to p53-RNA interactions are interesting: although recombinant p53 protein binds RNA in a sequence-nonspecific mode [65], RNA binding is prevented by post-translational p53 modifications [66], again suggesting that phosphorylation alters the distribution of the ensemble. An insight into the effect of protein factors on the differential activation of p53 target genes was also recently provided, particularly into the CDK-module of the human Mediator complex which functions as stimulus-specific positive coregulators of p21 transcription [67]. These manifest the complexity of the p53-related cellular pathways and in particular, how evolution has taken advantage of its rugged energy landscape illustrating that ruggedness away from the native state has a functional role [59],[68].

In summary, tumor suppressor p53 elicits cooperative binding with its response elements through efficient p53 core domain dimer-dimer interactions. This occurs when the response elements contain small base pair spacers between the two half sites. For p53-REs with more than three bp spacers, cooperativity is very low or needs involvement of other cellular components. These results, combined with broad genomic studies of p53-REs which revealed that the majority of the p53REs have 0–1 base pair insertions [7],[9] lead us to propose that the genomic organization of (most) functional p53REs is dominated by the need for cooperative interactions. This result is not surprising: cooperativity is well known to be a key player in biology.

## Methods

### MD simulation protocol

MD simulations were performed on the Shakked group crystal structure [30] and on four models with p53-REs containing 1, 2, 9 and 10 bp insertions. Each system was solvated with a TIP3P water box [69] with a margin of at least 10 Å from any edge of the water box to any protein or DNA atom. Solvent molecules within 1.6 Å of the DNA or within 2.5 Å of the protein were removed. The systems were then neutralized by adding sodium ions. The resulting systems were subjected to a series of minimizations and equilibrations using the CHARMM program [70] and the CHARMM 22 and 27 force field for the protein and nucleic acid, respectively [71]. Each system was minimized 1000 steps with the steepest descent algorithm and 1000 steps with the ABNR algorithm. For the second set of the simulations of the insertions 9 and 10 complexes, an additional 1000 steps of steepest descent minimization were applied to generate the slightly different configuration as the starting structures. The production MD simulations were performed at temperatures of 300 degrees Kelvin using the NAMD program [72] and the CHARMM force field. Periodic boundary conditions were applied and the non-bonded lists were updated every 20 steps. NPT ensemble was applied and the pressure kept at 1 atom using Langevin-Nose-Hoover coupling. SHAKE constraints [73] on all hydrogen atoms and a time step of 2 fs and a nonbonded cutoff of 12 Å were used in the trajectory production with a spherical shift function. This cutoff scheme was shown to perform well in both protein and DNA systems [74],[75]. The sizes of the systems were about 110,000 atoms and the duration for each simulation was 60 ns.

### p53 core domain dimer-dimer interface search

The crystal structure [30] was again used and the two p53 core domain dimer-DNA motifs were extracted from structure I in the crystal structure file. The purpose was to examine all possible p53 core domain dimer-dimer interfaces for p53-REs without bp insertions by changing the rotational angle of one p53 core domain dimer bound on DNA with respect to the other. The two base pairs, one from each DNA motif, that were in contact with each other in the crystal structure were not part of the p53-RE and therefore were removed. An axis was defined for each DNA segment as a line that passed through the centers of mass of the 2^nd^ and 3^rd^ base nucleotides at each end of the DNA duplex. The two modified p53-DNA complex motifs were then repositioned by merging the two DNA axis defined above. One DNA motif (along with its associated p53 dimer) was translated along the axis so that the two DNA segments were aligned to form a continuous 20-bp binding site. The same motif was rotated with respect to the axis to get a near B-form conformation at the interface of the two DNA motifs and ensure that the two p53 dimers were aligned to the same side of the DNA (Figure 2A, middle panel). One half site of the DNA together with its associated p53 dimer was rotated 10 degrees at a time with respect to the other half of the complex along the aforementioned DNA axis. Each resulting structure was then energy minimized for 10000 steps each with steepest Decent and ABNR algorithms. The minimized structures were then evaluated for the p53 dimer-dimer interaction energy and the structural features.

### Modeling of p53 dimer-dimer interfaces for complexes with base pair insertions

The p53 core domain dimer-half site DNA complex generated previously [17] using the crystal structure of Cho et al [14] was used for the construction of all complexes with bp insertions. The advantage of using this crystal structure is that Arg248 was positioned in the minor groove and therefore maintained extensive contacts with the DNA. Residues Arg180 and Glu181 were well positioned to form the salt bridges that are important to the dimerization in the DNA bound state while in other crystal structures Arg248 anchored at the DNA surface and only touched backbone of one DNA chain. Other structures also lack the salt bridging interactions between Arg180 and Glu181 [30],[31]. For 1 and 2 bp insertions, the two pre-constructed p53 core domain dimers were superimposed onto a canonical DNA template with alternative A and T base sequence so that the two half sites would be separated by one or two base pairs with the sequence of T and AT for the insertions, and so on. The template DNA was then removed except the spacer base pairs. The remaining DNA segments were covalently linked to obtain a continuous segment using the GENERATE module in the CHARMM program.

For insertions of 9 and 10 bps, pre-bent DNA segments were used. The generation of the pre-bent DNA was as follow: a 31-bp straight DNA duplex segment was forced to bend with the MMFP module in CHARMM by applying a force constant of 500 kcal/mol/Å^2^ between the centers of mass of base pairs 2–4 and 28–30 of the DNA; the distance was decreased by 0.25 angstrom at a time and 25-picosecond simulation was performed for each step to equilibrate the system. By matching the DNA in the p53 dimer-DNA complex with the bent DNA segments at various positions, a series of conformations with different p53 dimer-dimer organizations were generated. The conformations with maximum p53 dimer-dimer interactions from each series was selected and energetically minimized. The constructed models then were subjected to MD simulations to obtain the putative organization of the DNA-bound conformation. These two models were slightly adjusted by increasing the number of steps for minimization before the product MD simulations to obtain better p53 dimer-dimer interactions in the starting conformation and were used for the repeat simulations for both complexes with 9- and 10-base pair spacers.

## Supporting Information

Figure S1 p53 core domain tetramer-DNA complex models for p53-REs with 1–10 bp insertion. Canonical straight DNA was used in the construction of the models. Each model is illustrated in two orientations and the number of base pair insertions shown above each model.
(3.13 MB TIF)

Figure S2 p53 core domain tetramer-DNA complex model with two-bp insertion. (A) Starting structure conformation. (B) Final structure after the simulation. The data show that the p53 dimer (in red and cyan) rotated significantly anti-clockwise with respect to the other dimer. However, there was little contact between the dimers.
(7.24 MB TIF)

Figure S3 p53 core domain tetramer-DNA complex model with 9-base pair insertion. (A) Starting structure conformation. (B) Final structure after the simulation. (C) The atomic details of the dimer-dimer interface from the final structure. The residues at the interface are shown in different colors depending on their parent monomers. Residue pairs in close contact are indicated with dotted lines.
(8.06 MB TIF)

Figure S4 Structural and energetic changes from the second simulation of the complex with 9-base pair spacer. (A) p53 dimer-dimer interaction energy. (B) p53-DNA interaction energy. (C) The slightly modified starting structure and the average structure from the final 5 ns trajectory.
(3.85 MB TIF)

Figure S5 Structural and energetic changes from the second simulation of the complex with 10-base pair spacer. (A) p53 dimer-dimer interaction energy. (B) p53-DNA interaction energy. (C) the distance between the centers mass for the two pairs of p53 core domain for one and ten bp insertion complexes, respectively. The interacting p53 core domain pairs were the same as defined in Figures 4 and 5. (D) The slightly modified starting structure and the average structures from different segements of the trajectory.
(4.68 MB TIF)
