# Supplementary figures and images for: Cooperativity Dominates the Genomic Organization of p53-Response Elements: A Mechanistic View
Source: PLoS Comput Biol. 2009 Jul 24;5(7):e1000448. doi: 10.1371/journal.pcbi.1000448 (PMC2705680; doi:10.1371/journal.pcbi.1000448)

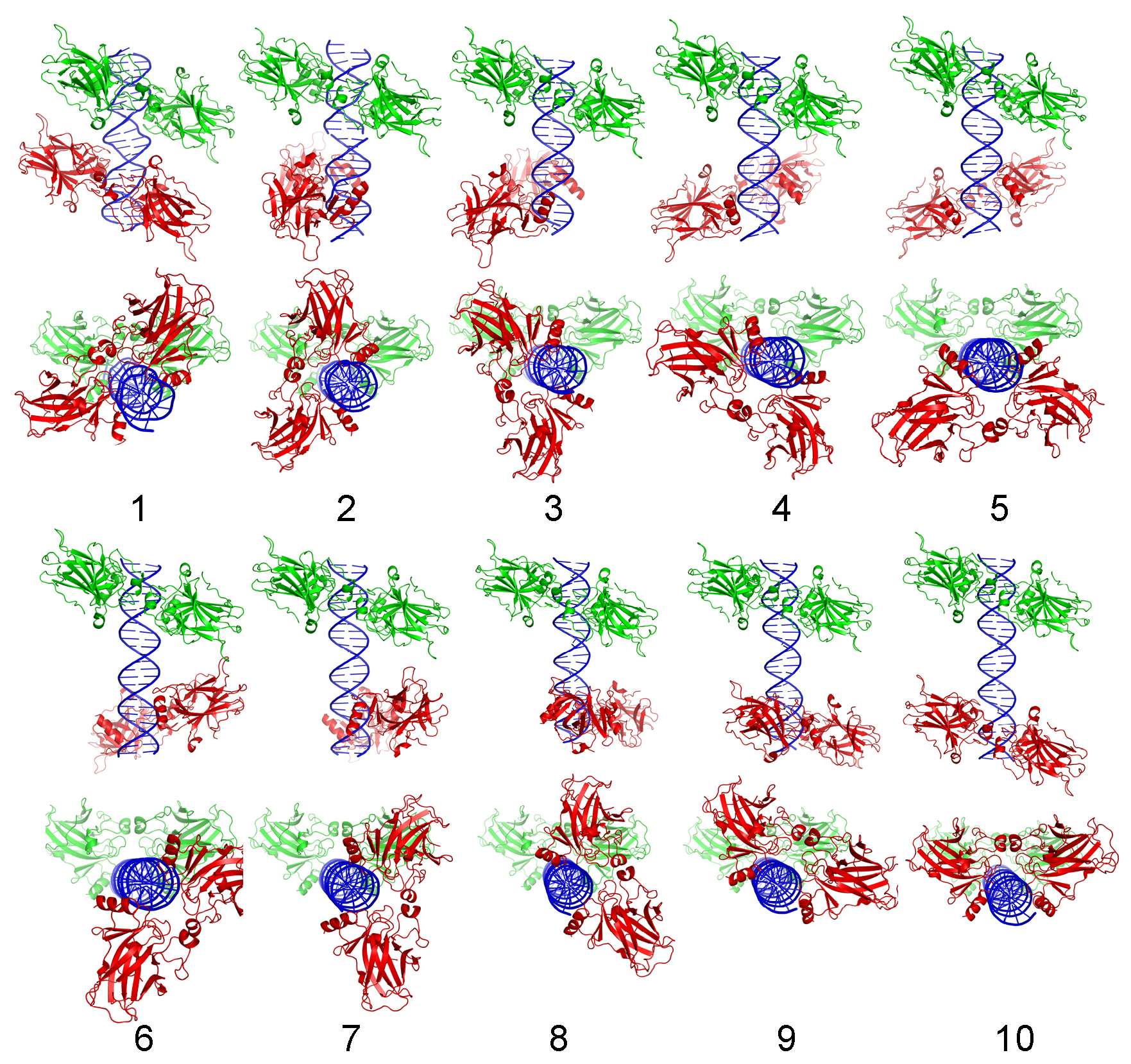

Supplement: Figure S1 — p53 core domain tetramer-DNA complex models for p53-REs with 1–10 bp insertion. Canonical straight DNA was used in the construction of the models. Each model is illustrated in two orientations and the number of base pair insertions shown above each model. (3.13 MB TIF) [file pcbi.1000448.s001.tif]

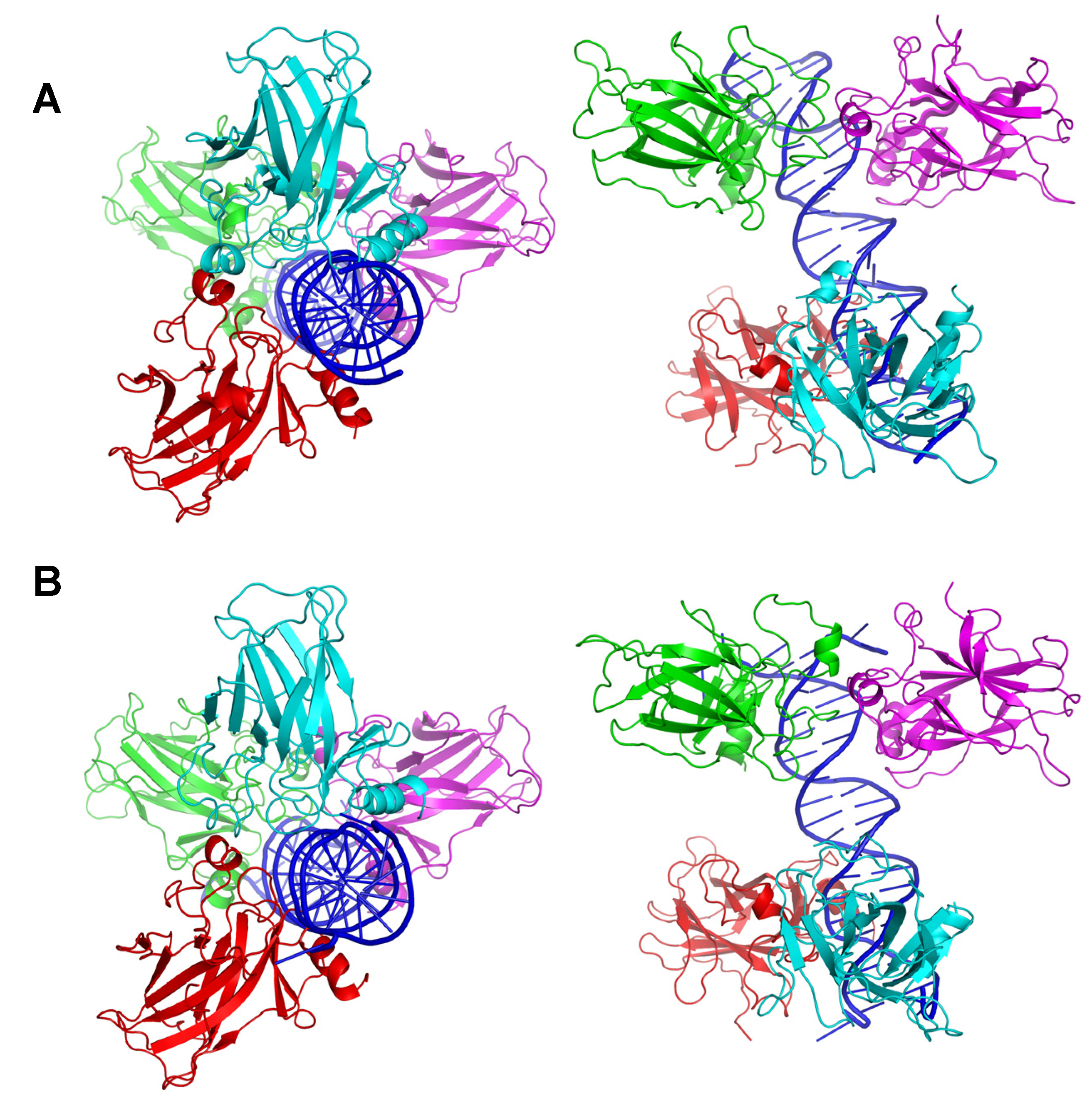

Supplement: Figure S2 — p53 core domain tetramer-DNA complex model with two-bp insertion. (A) Starting structure conformation. (B) Final structure after the simulation. The data show that the p53 dimer (in red and cyan) rotated significantly anti-clockwise with respect to the other dimer. However, there was little contact between the dimers. (7.24 MB TIF) [file pcbi.1000448.s002.tif]

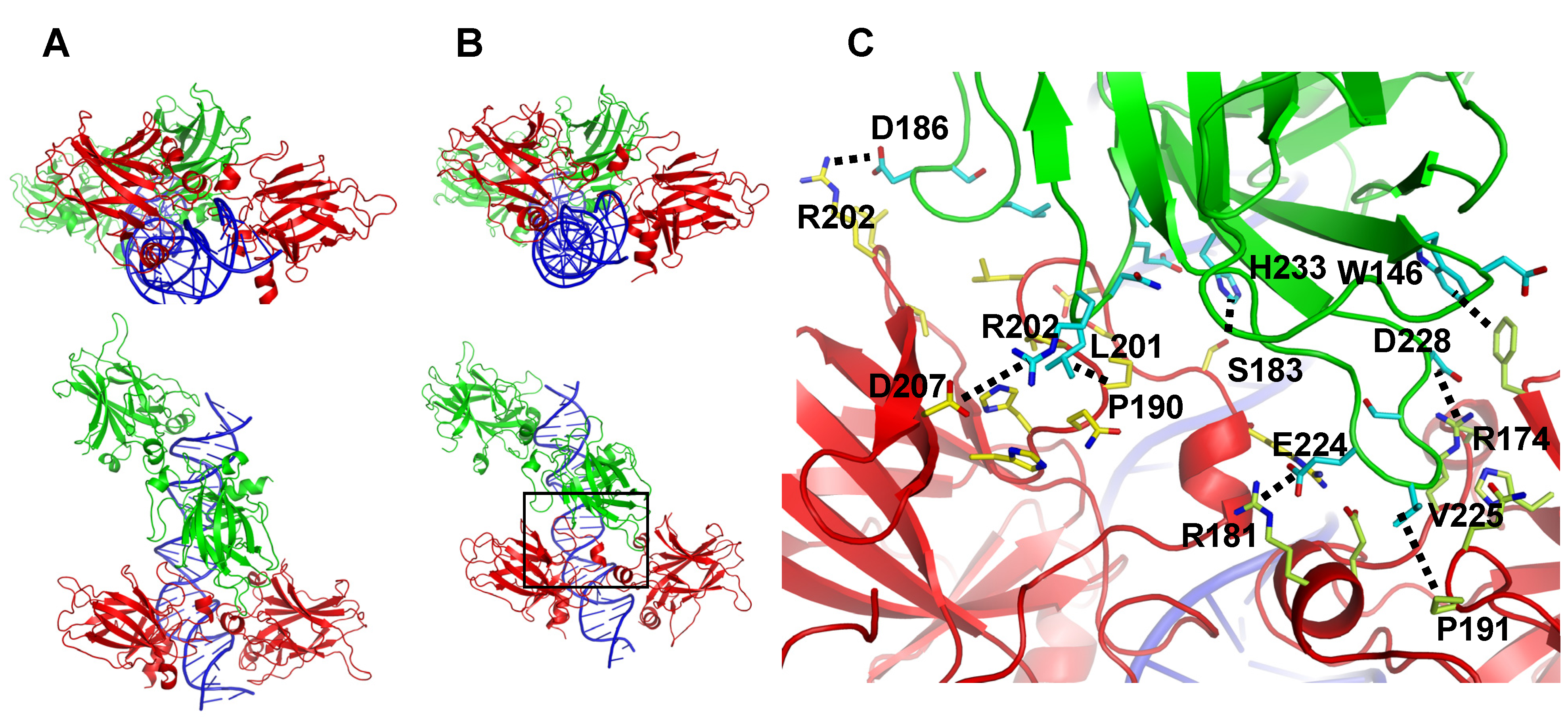

Supplement: Figure S3 — p53 core domain tetramer-DNA complex model with 9-base pair insertion. (A) Starting structure conformation. (B) Final structure after the simulation. (C) The atomic details of the dimer-dimer interface from the final structure. The residues at the interface are shown in different colors depending on their parent monomers. Residue pairs in close contact are indicated with dotted lines. (8.06 MB TIF) [file pcbi.1000448.s003.tif]

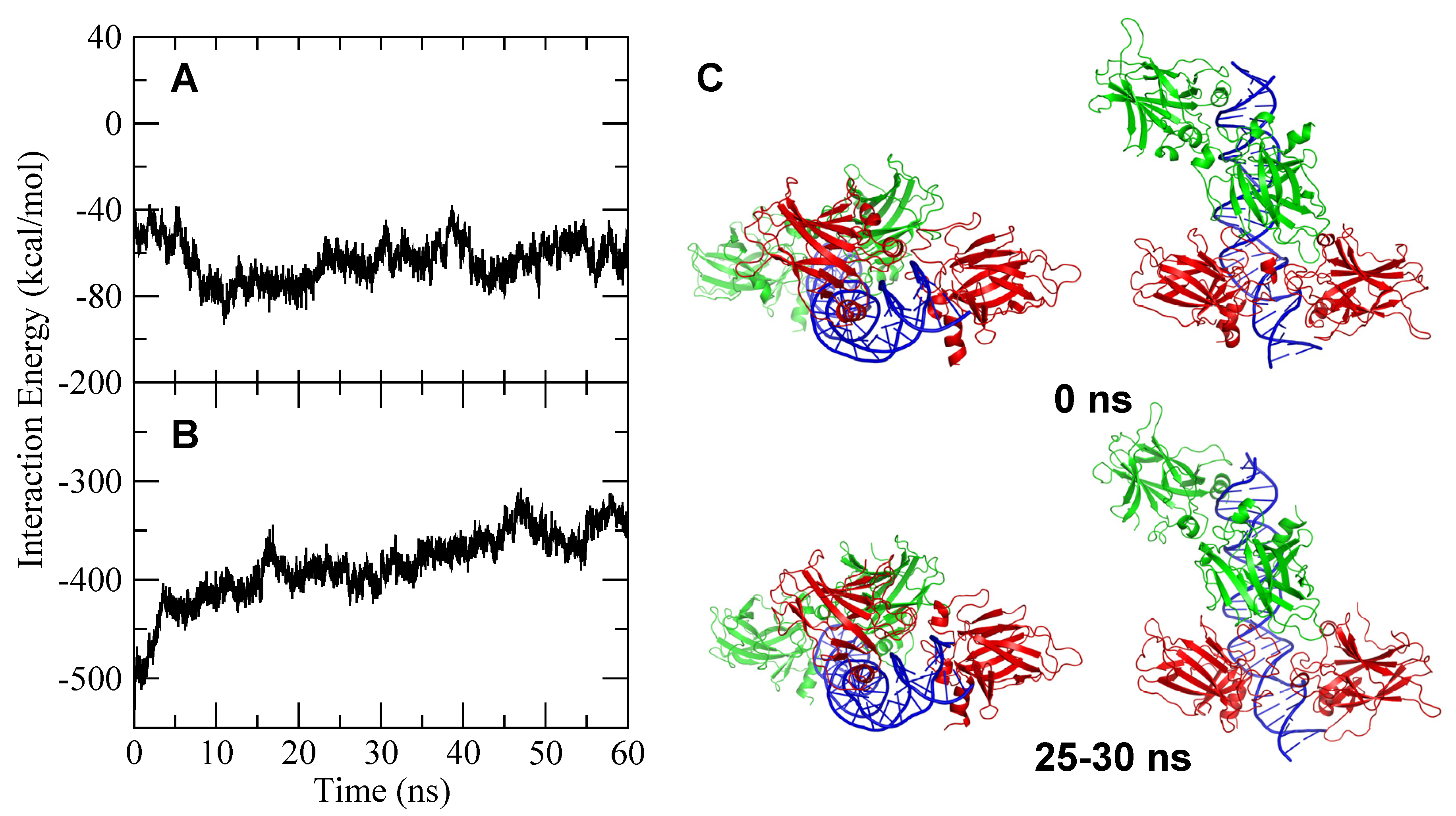

Supplement: Figure S4 — Structural and energetic changes from the second simulation of the complex with 9-base pair spacer. (A) p53 dimer-dimer interaction energy. (B) p53-DNA interaction energy. (C) The slightly modified starting structure and the average structure from the final 5 ns trajectory. (3.85 MB TIF) [file pcbi.1000448.s004.tif]

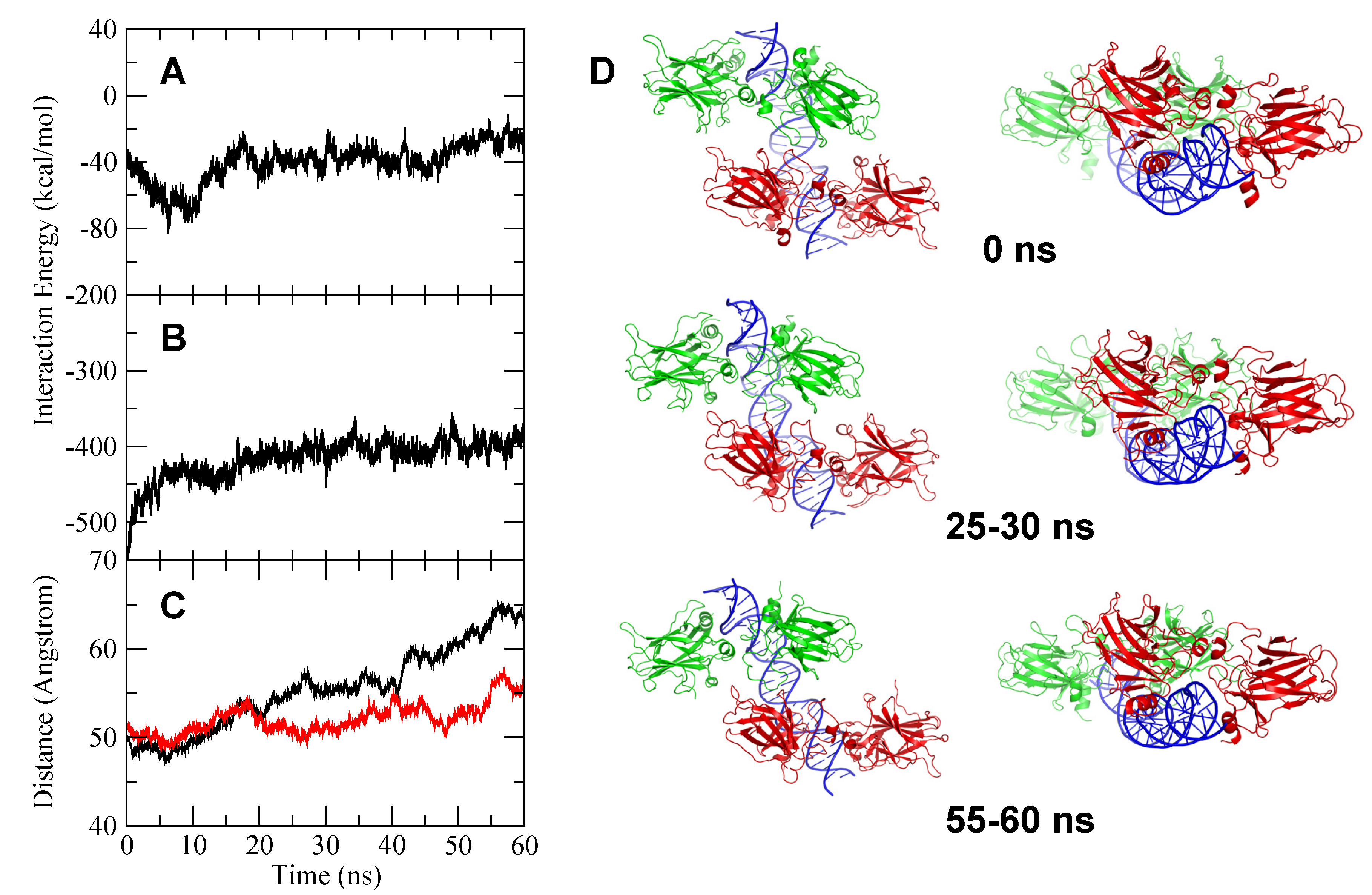

Supplement: Figure S5 — Structural and energetic changes from the second simulation of the complex with 10-base pair spacer. (A) p53 dimer-dimer interaction energy. (B) p53-DNA interaction energy. (C) the distance between the centers mass for the two pairs of p53 core domain for one and ten bp insertion complexes, respectively. The interacting p53 core domain pairs were the same as defined in Figures 4 and 5. (D) The slightly modified starting structure and the average structures from different segements of the trajectory. (4.68 MB TIF) [file pcbi.1000448.s005.tif]
